# Current status of single port robotic-assisted reconstructive urology: a systematic review, meta-analysis and structured summary of the available literature

**DOI:** 10.1007/s11701-025-02509-9

**Published:** 2025-07-04

**Authors:** Valerio Santarelli, Fabio Maria Valenzi, Hakan Bahadır Haberal, Luca A. Morgantini, Arianna Biasatti, Muhannad Aljoulani, Filippo Carletti, Alexandru Turcan, Flavia Tamborino, Francesco Del Giudice, Stefano Salciccia, Alessandro Sciarra, Simone Crivellaro

**Affiliations:** 1 Department of Maternal-Infant and Urological Sciences, “Sapienza” Rome University, Policlinico Umberto I Hospital, 00185 Rome, Italy; 2 Department of Urology, University of Illinois at Chicago, Chicago, IL USA; 3 Urology Unit, Department of Medico-Surgical Sciences and Biotechnologies, Faculty of Pharmacy and Medicine, Sapienza University of Rome, 04100 Latina, Italy; 4 Department of Urology, Ankara Ataturk Sanatoryum Training and Research Hospital, Ministry of Health, University of Health Sciences, Ankara, Turkey; 5 Urological Clinic, Department of Medicine, Surgery and Health Sciences, University of Trieste, 34149 Trieste, Italy; 6 Department of Urology, Humanitas Research Hospital-IRCCS, Rozzano, Italy; 7 Department of Biomedical Sciences, Humanitas University, Pieve Emanuele, Italy; 8 Department Surgery, Oncology and Gastroenterology, Urologic Unit, University of Padova, 35122 Padua, Italy; 9 Department of Oncology, Division of Urology, University of Turin, San Luigi Gonzaga Hospital, 10043 Orbassano, Italy; 10 Department of Medical Oral and Biotechnological Science, Università degli Studi “G. d’Annunzio” of Chieti, 66100 Chieti, Italy

**Keywords:** Reconstructive urology, Single Port, Robotic Surgery, Systematic Review, Meta-Analysis, Pyeloplasty

## Abstract

Single Port (SP) surgery is the most relevant surgical innovation of the past six years. Few data are available on the advantages and disadvantages of SP Robotic-Assisted reconstructive urology. In the present systematic review, we summarize the results of available literature exploring the feasibility of SP reconstructive urological procedures and comparing the SP and Multi Port (MP) approaches. The MEDLINE, EMBASE and Cochrane Library Databases were systematically searched for articles evaluating outcomes of SP robotic-assisted pyeloplasty, ureteral reimplantation, Boari Flap, fistula repair, bladder diverticulectomy and vaginoplasty. After meticulous study selection, we conducted a meta-analysis to compare perioperative and postoperative outcomes of SP and MP robotic-assisted pyeloplasty (SP-RP and MP-RP). Regarding ureteric reimplantation, Boari Flap, bladder diverticulectomy, fistula repair and vaginoplasty, we underwent a structured narrative synthesis, since meta-analysis was not feasible due to heterogeneity or insufficient study numbers. The meta-analysis included a total of six retrospective cohort studies and 202 patients. SP-RP demonstrated significantly lower Estimated Blood Loss (EBL) (SMD − 0.45, 95%CI − 0.80 to  − 0.09, *p* = 0.01, *I*^2^ = 0%) and better cosmetic results (MD 1.83, 95%CI 0.98–2.68, *p* < 0.001). The mean length of hospital stay was shorter for patients submitted to SP-RP, but the difference did not reach significance (SMD =  − 0.68, 95%CI − 1.43 to 0.07, *p* = 0.08, *I*^2^ = 80%). There were no significant differences in terms of complication rates, operative times, success rate and renal function increase between the two approaches (p > 0.05). Respectively, a total of four, one, two, one and two articles evaluating robotic-assisted SP ureteral reimplantation, Boari Flap, bladder diverticulectomy, fistula repair, and vaginoplasty, were included for the final structured summary. The included studies consistently suggest the feasibility and safety of the SP approach, however, available evidence predominantly consists of small retrospective series or individual case reports, and statistical validation is not possible. SP-RP has been successfully described, with possible advantages in terms of shorter hospital stays, better pain control and cosmetic results. Although successful cases of other major reconstructive urological procedures are reported in the literature, the available evidence remains limited and of low quality. Hopefully, the encouraging findings regarding SP-RP will increase the popularity of the SP robotic system among reconstructive urologic surgeons and the availability of more robust data.

## Introduction

Reconstructive urology is a subspecialty field of Urology that aims to restore physiologic urinary or sexual function by rerouting, repairing, or recreating specific parts of the genitourinary tract [1]. Conditions treated by reconstructive urologic surgeons are numerous and include trauma/injury, congenital disorders, acquired defects following stone formation, passage or management, as well as radiation or previous surgical treatments. Pyeloplasty, the standard treatment procedure for Ureteropelvic junction obstruction (UPJO) is the most performed urologic reconstructive procedure [2, 3]. Reconstructive urological interventions have been traditionally performed adopting an open approach. With the advent of minimally invasive surgery, a laparoscopic or robotic-assisted approach has become the standard for the major urological procedure [4]. Due to the generally younger population and the no of necessity for large skin incision to remove surgical specimens, minimally invasive reconstructive urology has become increasingly popular.

Most of the laparoscopic and robotic-assisted reconstructive urologic intervention are performed with a Multi Port (MP) approach. A Single Port (SP) approach has been proposed as an alternative to further reduce invasiveness and postoperative pain and improve cosmetic results [5]. In the case of LaparoEndoscopic Single Site Surgery (LESS), multiple laparoscopic instruments are inserted through a single incision. Despite its hypothetical advantages, the adoption of LESS has been limited by the difficult triangulation and inconvenient instruments’ crossing and conflict [6]. The da Vinci® (Intuitive surgical Inc., Sunnyvale, CA, USA) SP robotic system was developed specifically to perform single-site surgery and has been clinically available in the USA since 2018. The double-jointed instruments and the fully articulating 3DHD camera allow it to operate effectively in confined spaces without sacrificing maneuverability and reducing conflicts. Additionally, with the adoption of the SP robotic platform, the majority of urologic reconstructive interventions can be performed extra peritoneally and with the patient in a supine position, thus allowing simultaneous endoluminal instrumentation, reducing intraoperative complications, postoperative pain and ileus and ultimately allowing for a same-day discharge in the majority of cases [7, 8]. However, the more difficult instrument handling, narrower workspace and longer learning curve, when compared to the MP approach, are drawbacks that could potentially limit the widespread adoption of the SP robotic system in reconstructive urology, an already particularly challenging and demanding surgical subspecialty.

Despite its relatively novel introduction in the USA market and only one year since its EMA approval, the appeal of the SP robotic console is increasing, and literature is focusing on its accepted and plausible advantages. Articles exploring the safety and feasibility of SP reconstructive urologic surgery are available, but mostly in the form of case reports and small retrospective series. In the present study, we conducted a systematic review of reconstructive urology procedure in which the SP robot has been currently applied. When statistically possible, a meta-analysis was conducted to compare perioperative and postoperative outcomes of the MP and SP approaches. Alternatively, we opted for a structured summary of the relevant findings.

## Materials and methods

### Search strategy and study selection

The present systematic review and meta-analysis is registered under the following PROSPERO reference: CRD420251032891. We conducted a comprehensive literature review in adherence with the Preferred Reporting Items for Systematic Reviews and Meta-analyses (PRISMA) guidelines [9]. According to the Patient-Index-Comparator-Study (PICOS) design criteria [10], we established the following research question: What are the perioperative and postoperative outcomes of SP robotic-assisted main reconstructive urologic procedures and how these outcomes compare to those of the MP counterparts? The procedures included were: Pyeloplasty, Ureteric reimplantation, Boari Flap, vesicovaginal or enterovesical fistula repair, augmentation cystoplasty and bladder diverticulectomy. Two authors (VS and FMV) undertook independent and systematic search of major databases (MEDLINE, EMBASE and Cochrane). Search results were narrowed to studies written in English and published between 2018 (year of FDA approval of the first single-port robotic platform) and March 2025. Both the Title/Abstract/keywords and the Subject Headings section were searched. The search terminology was the following: (“single site” OR “single port” OR “single hole”) AND (“robot-assisted” OR “robotic-assisted OR “robotic*” OR “robot”) AND ( “pyeloplasty” OR “ureteropelvic junction”/“ureteral reimplantation” OR “ureteral reimplant”/“Boari Flap”/“bladder diverticulum” OR “bladder diverticula” OR “diverticulectomy”/“cystoplasty” OR “augmentation cystoplasty” OR “bladder augmentation/“fistula repair” OR “vesicovaginal fistula” OR “enterovesical fistula” OR “rectourethral fistula” OR “urinary tract fistula”/“vaginoplasty”). Conference abstracts, letters to the editor, reviews were excluded.

### Meta-analysis of SP vs. MP robotic-assisted pyeloplasty

#### Data extraction and quality assessment

Prospective or retrospective cohort studies, randomized controlled trials, comparing robotic-assisted MP and SP pyeloplasty were included for further analysis. Two authors (VS and FMV) independently and meticulously collected data from pertinent studies, including demographic data, estimated blood loss (EBL), operative time (OT) length of hospital stay (LOS), postoperative pain scores, complication rates, success rates, cosmetic results and estimated glomerular filtration rate (eGFR) changes. Radiological improvement of hydronephrosis and the disappearance of flank pain defined surgical success. When continuous data was presented using median values and ranges, predetermined statistical techniques were adopted for conversion to Mean and Standard Deviation (SD) [11]. The two authors independently evaluated the quality of the included studies on the basis of the modified version of the Downs and Black tool [12]. In the case of a disagreement, it was resolved through discussion with a third author. Any missing necessary information from the available studies was retrieved by contacting the corresponding author for additional details.

#### Statistical analysis

Continuous and dichotomous variables were compared using, respectively, standardized mean difference (SMD) and risk ratio (RR) with a 95% confidence interval (95% CI). A *p* value < 0.05 defined statistical significance. Heterogeneity was assessed using the Chi-square-based Q test and statistic *I*^2^. In cases of a significant heterogeneity, as indicated by an *I*^2^ value > 50% and a *p* value < 0.05, the pooled effect was evaluated using a random-effects model following the DerSimonian and Laird method [13]. Otherwise, the pooled effect was determined using a fixed-effects approach with the Mantel–Haenszel technique [22]. Begg’s funnel plot and Egger’s test were employed for publication bias assessment. Review Manager version 5.4 was adopted to conduct statistical analysis.

### Structured summary of other included reconstructive urology procedures

Regarding ureteric reimplantation, Boari Flap, bladder diverticulectomy, fistula repair and vaginoplasty, current literature is scarce and a meta-analysis or other statistical synthesis methods was not possible due to the excessive risk of bias. Under these circumstances, in accordance with recommendations for systematic reviews with limited data, we opted for a structured narrative synthesis with tabulation of results of individual studies, to efficiently and transparently report the available results.

## Results

### Pyeloplasty

Ureteropelvic junction obstruction (UPJO) is diagnosed in roughly 20% of infants with prenatal hydronephrosis [2]. The natural history of the disease is extremely variable, ranging from completely asymptomatic to recurrent flank pain, renal stones, pyelonephritis, and renal function loss [2, 14]. For diagnosis and follow-up in adults, Computerized Tomography (CT) Urogram and Renal Scintigraphy (RS) are the imaging modalities of choice [15]. Pyeloplasty is the standard treatment procedure. Due to the extreme variability of the condition, as well as the lack of clear treatment guidelines, the timing of surgery can vary between infancy and late adulthood [3]. Traditionally, pyeloplasty has been performed with an open approach. However, in recent years, the advantages of laparoscopic and robotic-assisted pyeloplasty have become clear [16, 17].

#### Study selection and characteristics of studies

**Fig. 1:**
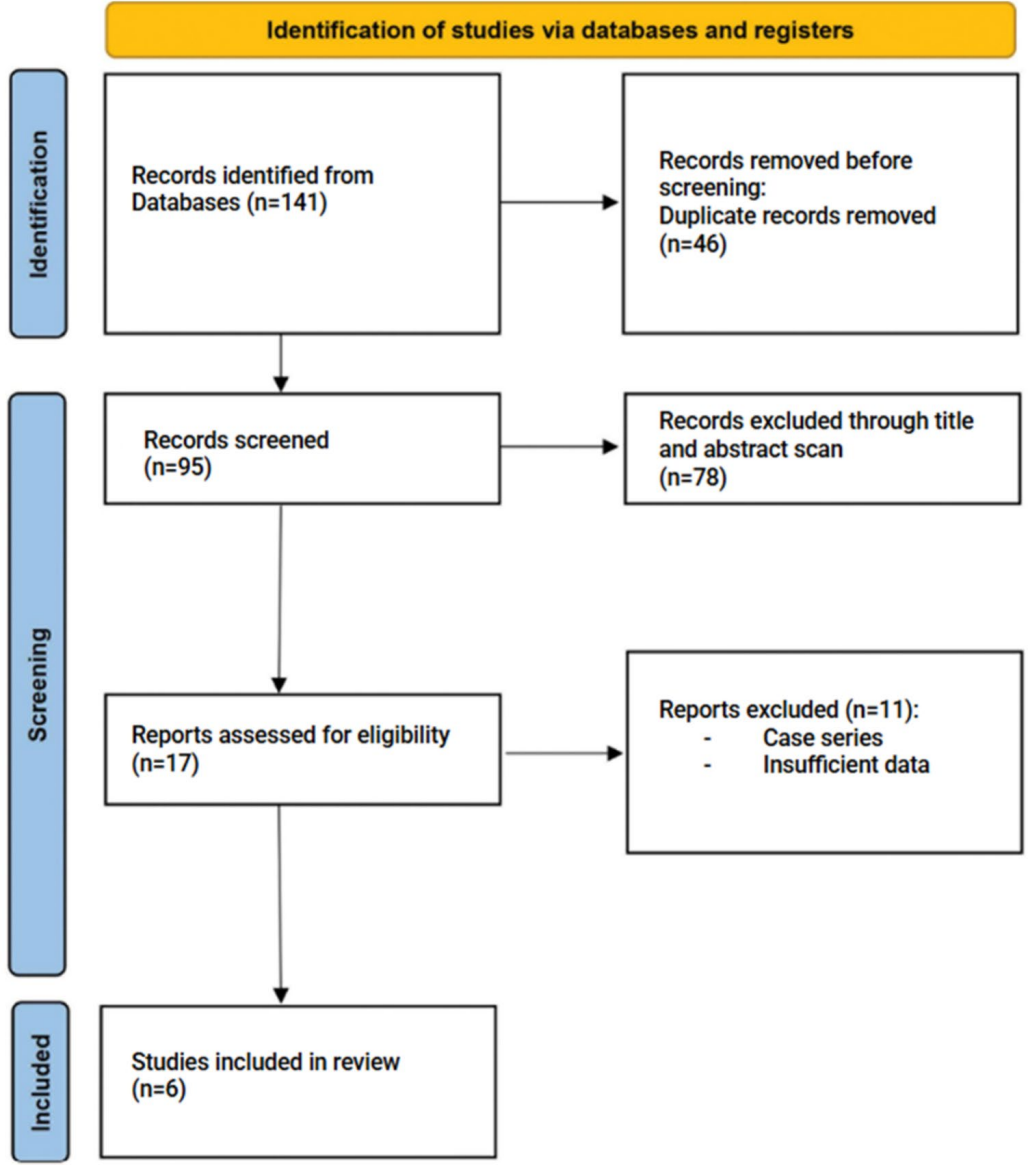
Flow diagram of selection of eligible studies

The selection processes adopted to choose relevant literature for the present study are depicted in Fig. 1. A total of 141 articles were initially found using the year filters and search methods previously discussed. After duplicate exclusion and subsequent selection on the basis of title, abstract scan, and finally full-text scan, six articles comparing SP-RP with single-port platforms and MP-RP were finally included [18–23]. Selected studies are reported in Table 1. All the included articles were retrospective cohort studies. Four out of six studies focused on adults, while the other two concentrated on children. Comprehensively, 94 SP patients and 108 MP patients were included in the meta-analysis.

**Table 1:** Characteristics of the included studies comparing SP and MP robotic-assisted pyeloplasty

References	Year	Country	Age	SP patients, *n*	MP patients, *n*	Design	Operation years	Quality score
Beksac et al [18]	2021	USA	Adults	11	12	Retrospective	2018–2020	17
Harrison et al [20]	2021	USA	Adults	21	21	Retrospective	2019–2020	19
Kang et al [21]	2021	Korea	Children	15	31	Retrospective	2019–2020	18
Ditonno et al [19]	2023	USA	Adults	10	12	Retrospective	2021–2023	17
Smith et al [22]	2023	USA	Children	11	5	Retrospective	2021–2022	12
Heo et al [21]	2024	Korea	Adults	15	28	Retrospective	2016–2022	19

#### Surgical results—perioperative outcomes

**Fig. 2:**
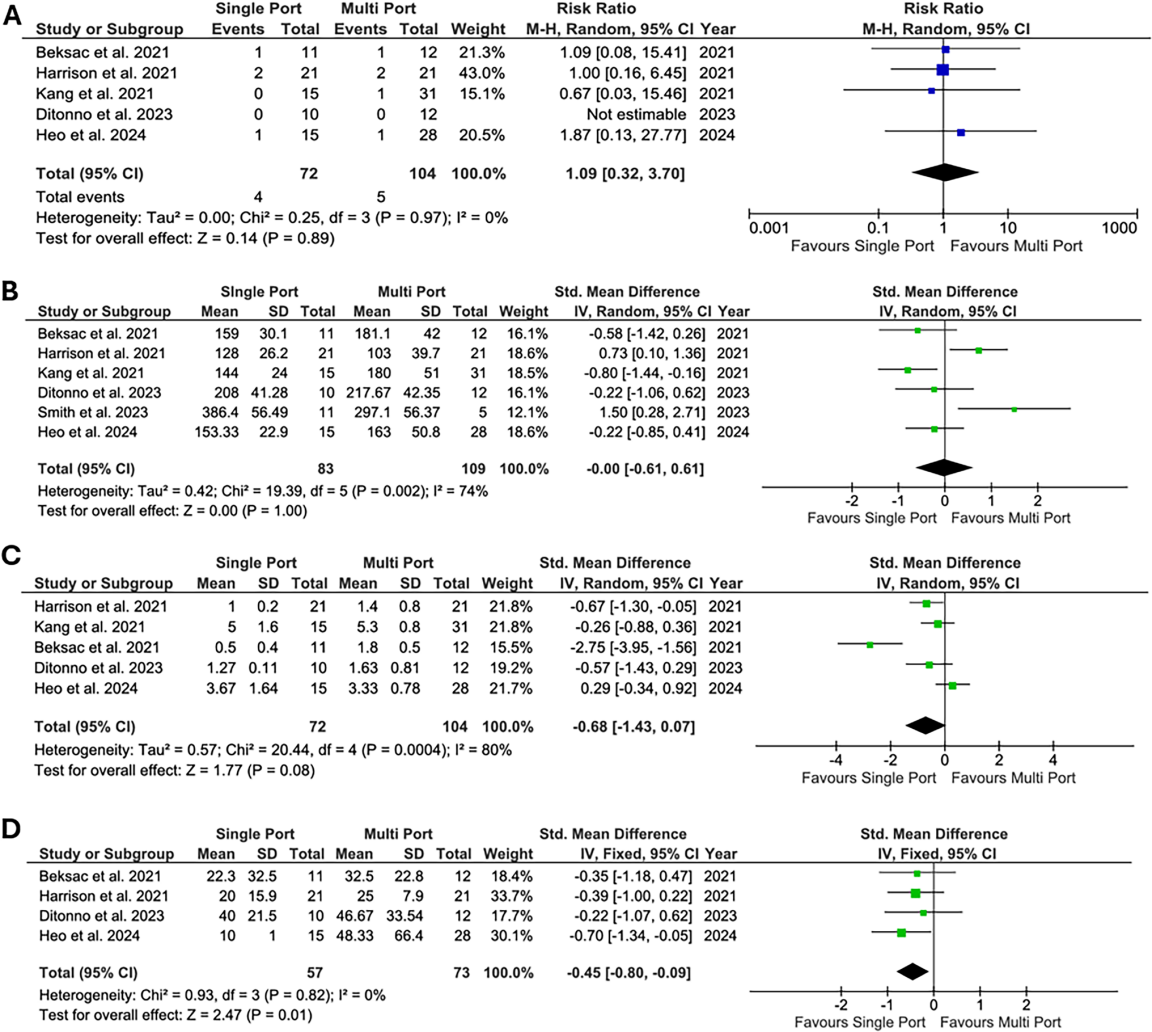
Forest plots depicting standardized mean difference or relative risk of perioperative outcomes. **A** Complications rate; **B** Operative time; **C** Length of stay; **D** Estimated blood loss (mL)

The results of the analysis on perioperative outcomes are shown in Fig. 2. SP-RP demonstrated superimposable complication rates (RR = 1.09, 95%CI 0.32–3.70, *p* = 0.89, *I*^2^ = 0% and OT (SMD =  − 0.00, 95%CI − 0.61 to 0.61, *p* = 1, *I*^2^ = 74%) of MP-RP. The mean LOS was shorter for patients submitted to SP-RP, but the *p* value was slightly over the predetermined level of significance when a random effect model was adopted (SMD =  − 0.68, 95%CI − 1.43 to 0.07, *p* = 0.08, *I*^2^ = 80%). EBL (mL) was lower in the SP-RP group compared to the MP-RP group (SMD − 0.45, 95%CI − 0.80 to  − 0.09, *p* = 0.01, *I*^2^ = 0%).

#### Surgical results—postoperative outcomes

**Fig. 3:**
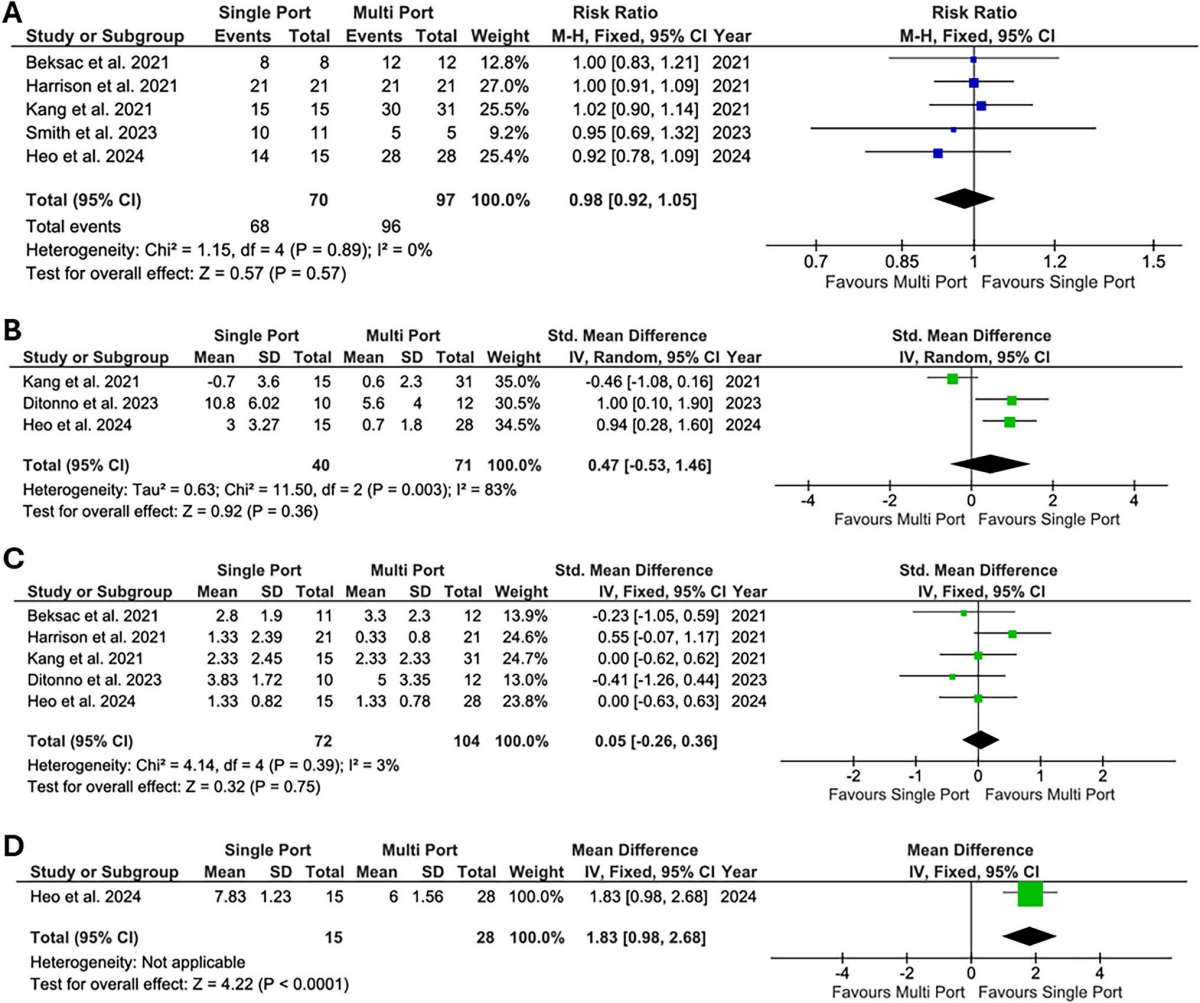
Forest plots depicting standardized mean difference or risk ratio of postoperative outcomes. **A** Success rate; **B** Renal function increase (ml/min); **C** Postoperative pain score; **D** Cosmetic result

Results of the analysis of postoperative outcomes are shown in Fig. 3. As previously reported, the radiologic improvement of hydronephrosis and the disappearance of flank pain were used to define surgical success. Success rate in the SP-RP group was not significantly inferior to that in the MP-RP group (RR = 0.98, 95%CI 0.92–1.05, *p* = 0.57, *I*^2^ = 0%). Renal function increase, expressed in mL/min increase in the eGFR, was comparable in the two groups (SMD 0.47, 95%CI − 0.53 to 1.46, *p* = 0.36, *I*^2^ = 83%). There was no significant difference in postoperative pain, determined by predetermined pain scores (SMD =  0.05, 95%CI -0.26 to 0.36, *p* = 0.05, *I*^2^ = 0%). Only one of the included studies evaluated cosmetic results, which resulted superior in the SP-RP group (MD 1.83, 95%CI 0.98–2.68, *p* < 0.001).

#### Publication bias

No substantial publication bias was discovered, as indicated by the absence of evident asymmetry in the Begg’s funnel plot and the > 0.05 result of Egger’s test *p* values for each of the outcomes of interest.

### Ureteral reimplantation

Ureteral reimplantation has a wide range of indications, mainly represented by vesicoureteral reflux, distal ureteral strictures, trauma, or iatrogenic injury. It is noteworthy that gynecologic surgery accounts for approximately 75% of ureteral injuries, with the incidence of this complication reported in up to 1.5% of such procedures [24]. Different techniques and approaches have been described to perform a ureteral reimplantation. Ultimately, the common goal of all of these techniques is to perform a tension-free and, when indicated, an anti-refluxive anastomosis. A robotic MP approach has demonstrated to reach similar outcomes to open surgery, with decreased morbidity and mortality [24–26].

#### Structured summary

Initial database screening adopting the search methods yielded a total of 23 studies. 4 articles were finally included after duplicate exclusion and further selection. The first to describe technique and outcomes of robot-assisted ureteric reimplantation using the da Vinci SP surgical system were Kaouk and colleagues in 2019 [27]. Authors analyzed an initial series of 3 cases. In all cases, surgery was performed via a single umbilical 2.5–3 cm incision through which the GelPOINT advanced access platform (Applied Medical, Rancho Santa Margarita, CA, USA) was placed for the insertion of the dedicated 25-mm SP multi-channel port. An additional 12-mm port was placed for the bedside assistant, either through the GelPOINT or through a separated incision. Authors reported a 100% success rate with 50 ml blood loss and no intraoperative or postoperative complications in any of the reported cases. A comparison of the robotic-assisted SP technique with the standard MP robotic-assisted ureteral reimplantation was performed by Lin et al. in 2024. However, the SP procedures, despite being carried out via a single incision, were not performed with a dedicated SP robot, but instead using an MP robotic platform [28]. 24 pediatric patients (12 in the SP and 12 in the laparoscopic cohorts) diagnosed with primary obstructive megaureter were retrospectively evaluated. All procedures were successful. The SP robotic cohort demonstrated slightly longer operative time, but significantly lower blood loss and shorter hematuria, catheterization and hospitalization time. The same year Chen et al. compared outcomes of 13 SP and 19 MP robotic-assisted ureteral reimplantation performed on pediatric patients with vesicoureteral reflux [29]. However, similarly to the previous paper, the SP cases were performed adapting the MP surgical console to a SP procedure. Not surprisingly, authors found comparable results between the two techniques. An initial case series to evaluate the feasibility and safety of ureteral reimplantation using the SP robotic system was published by Heo et al. in 2023 [30]. 21 patients diagnosed with distal ureteral stricture who underwent SP robotic-assisted ureteral reimplantation were analyzed. Many patients had undergone previous abdominal surgery. 100% of patients reached radiographic and symptomatic improvement after 3 months. However, 61.9, 4.8 and 9.5% of the cases required the insertion of an additional port, conversion to open surgery and had intraoperative complications, respectively. These results, while confirming the feasibility of urologic reconstructive urology using the SP system, suggest the possibility of an early consideration of an additional assistant port in patients who already underwent previous pelvic interventions resulting in significant adhesions (see Table 2).

**Table 2:** Characteristics of the included studies evaluating SP robotic-assisted ureteral reimplantation

Author and year	Study type	Surgical approach	Population, *n*	Population, age group	Outcomes
Kaouk et al., 2019 [27]	Case report	SP	3 SP	Adult	Successful without complications
Lin et al., 2024 [28]	Retrospective	SP vs. Lap	12 SP and 12 Lap	Pediatric	SP: Longer operative times but shorter hematuria, catheterization and hospitalization times
Chen et al., 2024 [29]	Retrospective	SP vs. MP	13 SP and 19 MP	Pediatric	Comparable results
Heo et al., 2023 [30]	Retrospective	SP	21 SP	Adult	100% radiographic and symptomatic improvement at 3 months

### Boari flap

A boari flap, a technique which utilizes a flap of bladder to reach a higher portion of the ureter, is a useful option when the diseased segment of the distal ureter is too long or ureteral mobility is limited. With this technique, a defect up to 15 cm in length can be repaired, compared to the 4–5 cm reached with a standard ureteral reimplantation. A precise description of the technique is provided by Stolzenburg et al.[31]. After access and colon mobilization, the ureter is first dissected distally to the site of stricture and subsequently clipped proximally to the diseased segment and transected caudally. The Boari flap is then created with a length-to-width ratio of 2–1 and a submucosal tunnel through which the spatulated ureter is pulled. Subsequently, the ureteral adventitia is sutured to the mucosa of the flap. A Double-J stent is then inserted and the flap tubularized. Finally, the bladder opening is closed. Despite promising results, even standard MP robotic data are limited. To date, the largest prospective, multi-institutional study available describing outcomes for patients undergoing Robotic-assisted ureteral reimplantation with a Boari Flap was published in 2023 [32]. Authors demonstrated low complication rates and high success rates of the 46 MP robotic-assisted Boari Flaps.

#### Structured summary

8 studies were found as a result of the initial search. After selection, only one study was included in the present review. Interestingly, the only available data on the feasibility of SP robotic-assisted Boari Flap come from the same aforementioned study evaluating MP Boari Flap [32]. Indeed, authors included 4 Boari Flaps performed with the SP robotic platform, through a single umbilical incision with an additional assistant port. All cases were successful and no complications were recorded. Additionally, the SP approach required shorter median operative times (215.0 min [IQR: 170.5–282.0]) and LOS (1 day [IQR: 1.0–1.5]) (see Table 3).

**Table 3:** Characteristics of the included studies evaluating SP robotic-assisted Boari Flap

Author and year	Study type	Surgical approach	Population, *n*	Population age group	Outcomes
Corse et al., 2023 [32]	Retrospective, multicenter	MP and SP	46 MP and 4 SP	Adult	100% success rate; SP: Shorter median OT and LOS

### Bladder diverticulectomy

Bladder diverticulum is defined as the outpouring of the mucosa through points of weakness of the muscular layer of the bladder. Diverticula caused by congenital defects of the detrusor layer are rare. The majority of cases are acquired and derive from conditions that increase intraluminal bladder pressure, the most frequent being benign prostatic hypertrophy (BPH) [33]. Complications associated with bladder diverticula are Urinary Tract Infections (UTIs), stone development, and cancer [33]. Diverticulectomy, performed with an open, laparoscopic or robotic-assisted approach, has been demonstrated to reduce post-void residual (PVR). It can be performed alone or in combination with a surgical treatment for Bladder Outlet Obstruction (BOO), either concomitant or staged [34]. A laparoscopic approach has been demonstrated to reduce postoperative morbidity and blood transfusions [35]. Davidiuk et al. reported their experience of 16 MP robotic-assisted bladder diverticulectomies performed by either an intravesical or extravesical approach [36]. The extravesical approach required longer operative times. No blood transfusions were required, the median LOS was 2 days and no 30-day postoperative complications of Clavien grade 3 or 4 were recorded. The median postvoid residual volume was decreased by more than half, improving from a pre-operative median of 458–214 mL (range, 46–527 mL) after surgery.

#### Structured summary

Successful SP robotic-assisted bladder diverticulectomy has been described, both via a transabdominal and transvesical approach, but only in the form of case reports. Accordingly, after article screening and selection, only 2 of the 9 evaluated studies were included [37, 38].

The two articles report a total of 3 cases, all successfully completed. Further larger series and prospective studies are required to evaluate the hypothetical advantages of a SP approach in this particular scenario (see Table 4).

**Table 4:** Characteristics of the included studies evaluating SP robotic-assisted bladder diverticulectomy

Author and year	Study type	Surgical approach	Population, *n*	Population age group	Outcomes
Gurung et al., 2020 [37]	Case report	SP EV and SP TV	1 SP EV and 1 SP TV	Adult male	Successful; EBL: 5 for EV and 100 for TV; LOS: 1 day
Tonzi et al., 2021 [38]	Case report	SP TV	1 SP TV	Adult male	Successful; EBL: 50 ml; LOS: 1 day

### Fistula repair

The most common fistulas affecting the urinary tract are rectourethral fistulas (RUFs), enterovesical fistulas (EVFs) and Vesicovaginal fistulas (VVFs). VVF is the most frequent and is mainly caused by iatrogenic causes, primarily gynecologic surgery. A conservative management is an option in selected cases. When surgical management is taken into consideration, the optimal treatment strategy is still uncertain. Different approaches (open, laparoscopic or robotic) and techniques (extravesical, transvesical or transvaginal) have been described in the available literature [39–42]. In particular, an MP robotic-assisted approach has been demonstrated to be feasible, with reduced blood loss and LOS when compared to an open approach [42]. RUF is a rare occurrence, but the risk increases in patients affected by inflammatory bowel diseases and perirectal abscesses. Moreover, RUF is a possible iatrogenic complication of radical prostatectomy (RP) and pelvic radiation, with an incidence of 1–11% after RP. RUFs can be repaired with different approaches, such as transvesical, transabdominal and transrectal, and more than 40 different techniques have been described. Despite a robotic-assisted approach being increasingly common in the last years, available data on robotic-assisted RUF repair is scarce and mostly in the form of case reports [43, 44].

#### Structured summary

Initial search yielded a total of 7 articles. After screening and selection, only one retrospective report of 4 robotic-assisted SP transvesical VVF repairs was included in the review. A preliminary cystoscopy was performed on patients in lithotomy position. After fistula detection, a 5fr stent was placed through the fistulous tract. Two ureteral stents were inserted. Subsequently, the patient was moved to a supine position and a transverse suprapubic 3 cm incision and 2 cm cystotomy were made for SP access. After dissection of the edges to reduce tension, a three-layer closure was performed: vagina, muscularis layer of the bladder and mucosal layer of the bladder. All fistulas were between 11 and 15 mm in diameter. In one case, the ureteral stents were not removed concomitant with the procedure but were left for two weeks, due to the proximity of the fistula to the ureteral orifices. One case developed a UTI. After a median follow-up of 8 months, no patients experienced recurrences [45]. Even though cases of SP robotic-assisted RUF repair have been presented, no article currently explores the feasibility of this technique (see Table 5).

**Table 5:** Characteristics of the included studies evaluating SP robotic-assisted VVF repair

Author and year	Study type	Surgical approach	Population, *n*	Population age group	Outcomes
Cannoletta et al., 2024 [45]	Case series	SP TV	4 SP TV	Adult female	Successful; 1 complication (UTI)

### Vaginoplasty

In recent years, the demand for gender affirming surgery is increasing and these interventions are being performed in patients with strong cross-gender identification. As gender affirming surgery, such as transgender vaginoplasty, are becoming more popular, evidence regarding its feasibility and safety is growing [46]. A combined open and robotic-assisted method is frequently adopted. While a perineal surgeon performs orchiectomy, penis degloving and perineal incision, using the urethra, neurovascular bundles, glans and corpora to perform a labiaplasty and clitoroplasty, the robot is docked and intraperitoneal space reached to create a posterior and anterior peritoneal flap, to be sutured to the inverted penoscrotal skin delivered by the perineal surgeon [47].

#### Structured summary

A total of 14 articles were initially found using the year filters and search methods previously discussed. After duplicate exclusion and subsequent selection, two articles evaluating both SP and MP robotic-assisted vaginoplasty were finally included. The first study demonstrating the feasibility of robotic-assisted vaginoplasty with peritoneal flap (Davydov technique) was carried out by Acar et al. on 11 consecutive patients (9 SP and 2 MP). With the combined approach, they were able to reach a mean postoperative vaginal depth of 13.9 ± 0.5 cm. The mean length of stay was 5.2 ± 0.6 days with a readmission rate of 18%. Only *n* = 1 patient (9%) required surgical revision of the neovagina [48]. In 2021, Dy et al. retrospectively compared the results of 53 SP and 47 MP Robotic-assisted peritoneal flap gender-affirming vaginoplasty (RPGAV)[49]. The average procedure time was shorter in the SP group (4.2 h vs 3.7 h, *p* < 0.001). Total complication rates were comparable between the two groups and generally low, with a 6, 1, 2, 1 and 7% rate of transfusion, rectovaginal fistula, bowel obstruction, pelvic abscess and vaginal stenosis, respectively. Average vaginal depth at a mean follow-up of 11.9 months was satisfactory (13.6 cm in the MP group and 14.1 cm in the SP group, *p* = 0.07). Results from this study suggest that, while both MP and SP approaches are feasible and safe, utilizing the SP robot can reduce the operative time since it facilitates a contemporaneous dual-surgeon abdominal-perineal approach (see Table 6).

**Table 6:** Characteristics of the included studies evaluating SP robotic-assisted vaginoplasty

Author and year	Study type	Surgical approach	Population, *n*	Population age group	Outcomes
Acar et. al., 2021 [48]	Retrospective	SP and MP	9 SP and 2 MP	Adult transgender	Successful; Readmission Rate: 18%; 1 surgical revision
Dy et al., 2021 [49]	Retrospective	SP vs. MP	53 SP and 47 MP	Adult transgender	Comparable results and complication rates; SP: Lower OT

## Discussion

Since its first FDA approval in 2018, the Intuitive DaVinci SP platform (Intuitive Surgical, Mountain View, California) has gained growing popularity among American urologic surgeons, especially in the reconstructive surgery field. After only a year since obtaining the CE mark, SP surgical robots are already becoming available around Europe. This novel technology aims to reduce invasiveness and complications associated with multiple incisions and transperitoneal access, while providing robotic surgeons a similar user experience to the standard MP consoles. Single-incision laparoscopic surgery is not a novel idea. A less approach has been proposed as a viable alternative to a standard laparoscopic approach, to reduce blood-loss and postoperative pain and increase cosmetic results [6]. Different studies have demonstrated the feasibility and safety of LESS pyeloplasty [50, 51]. Despite that, technical difficulties associated with the procedure have limited its widespread adoption. The SP robotic platform has been proposed to overcome these difficulties.

Results from our meta-analysis suggest that using a SP robotic platform does not prolong OTs of pyeloplasty. This is not an unexpected result, as also previous meta-analyses comparing SP and MP prostatectomies and nephrectomies also reported comparable OTs [52, 53]. In our analysis, SP robotic-assisted pyeloplasty (SP-RP) did not increase nor reduce the risk of complications. While other studies have reported a lower risk of complications with single-site robotic kidney surgery [53], obtaining the same statistical result for pyeloplasty is challenging, as it is a procedure with very low complication rates, and studies on very large cohorts would be required to obtain significance. Nonetheless, we found a lower mean EBL in SP-RP patients. We reported lower mean LOS with the SP approach, but the difference was slightly over the level of significance. Despite that, it is not hard to imagine shorter hospital stays of patients operated with the SP platform, thanks to the single incision, supine position, extraperitoneal access (when feasible and preferred) and lower opioid use that mostly allow for a same-day discharge in an outpatient setting [54]. In addition, a small, single incision and the supine position also aid in reducing postoperative pain and consequently allowing a quicker discharge and an opioid-free remission.   In light of the single incision required to perform SP-RP, better cosmetic results are an evident advantage over MP robotic-assisted pyeloplasty (MP-RP). However, of the included studies, only Heo et al. evaluated satisfaction with cosmetic appearance, demonstrating higher cosmetic appearance scores of SP-RP over MP-RP [21]. Routinely, SP-RP was performed through a 2–3 cm umbilical incision [19, 20, 22, 23, 55]. Only in the Beksac et al. study and the initial cases of Smith et al. was the intervention carried out through a single ∼3 cm incision above the pubic bone and lateral to the midline [18, 22]. As reported by Heo et al., it appears that the closeness to the umbilical region helped mask the surgical scar [21]. This is one of the first meta-analysis comparing MP-RP with SP-RP using the novel SP robotic platform. However, our study is not without limitations. First, while our study cohort is the largest available comparing SP-RP with MP-RP, the sample size is still relatively small. Secondly, not all the explored results were available in every included study. Lastly, only retrospective studies of moderate quality were available for inclusion, increasing the risk of blindness and distribution biases.

Beyond the meta-analyzed pyeloplasty data, the structured summaries provide insight into the state-of-the-art adoption of the SP platform across various reconstructive procedures. The included studies consistently suggest feasibility and safety of the SP approach, however, they were mostly in the form of small retrospective series or isolated case studies. These types of publications are generally more prone to potential biases, mainly publication bias favoring positive outcomes, selection bias towards simpler cases, and lack of a comparator and long-term follow-up. Moreover, while for the meta-analyzed pyeloplasty we were able to select only articles reporting procedures performed with the novel SP robotic platform, some “SP” procedures included in the structured summaries were performed adapting the MP system to SP access. However, these favorable preliminary findings might encourage reconstructive urologic surgeons to consider SP approaches to increase the availability of more robust and comparative data.

## Conclusions

Reducing invasiveness is one of the main goals of surgical innovation. The rationale behind single-site surgery in reconstructive procedures, mostly performed on young patients and without the limitations of specimen removal, is even clearer. Nonetheless, due to the relative rarity of such procedures, data on outcomes of SP robotic-assisted urologic reconstructive surgery are limited. Results from our systematic review and meta-analysis suggest SP-RP to be a safe and technically achievable procedure, with possible advantages over the MP approach, in terms of lower blood loss, shorter hospital stays, and better cosmetic results. Regarding other major reconstructive urological procedures, results of our systematic review and structured summary suggest an initial interest of robotic surgeons towards an SP approach. However, the scarcity of available data, in the form of small retrospective series or individual case reports, does not allow for any significant conclusions to be drawn. With the increasing international diffusion of the SP surgical system, higher-quality evidence will hopefully be available in the near future.

## Data Availability

Data is provided within the manuscript or supplementary information files.
